# Combination with Continual Learning Update Scheme for Power System Transient Stability Assessment

**DOI:** 10.3390/s22228982

**Published:** 2022-11-20

**Authors:** Bowen Hu, Zhenghang Hao, Zhuo Chen, Jing Zhang

**Affiliations:** Department of Electrical Engineering, Guizhou University, Guiyang 550025, China

**Keywords:** continual learning, transient stability assessment, model update, network topology change, deep residual shrinkage network

## Abstract

In recent years, the power system transient stability assessment (TSA) based on a data-driven method has been widely studied. However, the topology and modes of operation of power systems may change frequently due to the complex time-varying characteristics of power systems. which makes it difficult for prediction models trained on stationary distributed data to meet the requirements of online applications. When a new working situation scenario causes the prediction model accuracy not to meet the requirements, the model needs to be updated in real-time. With limited storage space, model capacity, and infinite new scenarios to be updated for learning, the model updates must be sustainable and scalable. Therefore, to address this problem, this paper introduces the continual learning Sliced Cramér Preservation (SCP) algorithm to perform update operations on the model. A deep residual shrinkage network (DRSN) is selected as a classifier to construct the TSA model of SCP-DRSN at the same time. With the SCP, the model can be extended and updated just by using the new scenarios data. The updated prediction model not only complements the prediction capability for new scenarios but also retains the prediction ability under old scenarios, which can avoid frequent updates of the model. The test results on a modified New England 10-machine 39-bus system and an IEEE 118-bus system show that the proposed method in this paper can effectively update and extend the prediction model under the condition of using only new scenarios data. The coverage of the updated model for new scenarios is improving.

## 1. Introduction

With the rapid development of modern power systems, increasing penetration of renewable energy sources and power electronics, as well as the expanding scale of power systems with regional interconnections, the power system is running closer to its stability limits [1]. When the power system is disturbed, the problems of transient instability are more likely to occur, which is an influential trigger for large-scale blackouts in the grid [2,3,4]. Thus, a fast and accurate method of transient stability assessment is essential for the safe and stable operation of power systems [5].

The current methods for transient stability assessment include time-domain simulation methods [6,7], direct methods [8,9], and data-driven methods [10]. The time-domain simulation method can model the system in detail with high accuracy of calculation, which is time-consuming and difficult to apply online. The direct method is hard to evaluate accurately and reliably with its highly simplified model and has poor adaptability facing large and complex grids. In recent years, with the continuous development of the synchronous phase measurement unit (PMU) [11] technology and the ongoing improvement of the wide-area measurement information system (WAMS) [12]. The PMU installed in the grid can obtain a large amount of system dynamic information simultaneously, providing a powerful data foundation for the data-driven approach. The method constructs a mapping relationship between transient stability data and stability conclusions, which enables fast and accurate transient stability assessment without the need to build complex mathematical models. At present, there are numerous research results based on data-driven TSA, among which shallow machine learning algorithms mainly involve Support Vector Machines (SVM) [13], Decision Trees (DT) [14], Random Forests (RF) [15], Artificial Neural Networks (ANN) [16], etc. Since they have limited capabilities in data mining, they have inadequate generalization capabilities when dealing with complex problems. As the accelerated advancement of data-driven technology, it provides new ideas for power system transient stability assessment by deep learning with stronger learning mining capability. Such as Deep Belief Networks (DBN) [17], Convolutional Neural Networks (CNN) [18,19], Long Short-Term Memory Networks (LSTM) [20], etc. The proposed evaluation framework in [18] is capable of performing both TSA as well as instability mode prediction. The cascaded CNN built in [19] obtains safe and reliable evaluation results by continuously increasing the amount of fault information contained in the input. A hierarchical deep learning machine is designed in [21] to perform transient stability assessment in both quantitative and qualitative terms. In [22,23] considering the connection relation of the network, a graph convolutional network (GCN) is used to learn spatial information to construct Spatio-temporal features to achieve an effective TSA. In [24] a generative adversarial network (GAN) is introduced, specifically for dynamic safety assessment of systems with missing data.

In the above studies, it is usually assumed that the training data follow the same data distribution as the online monitoring data. However, in actual operation, new scenarios will emerge from the system that were not considered in the TSA database for maintenance control measures and natural disasters. When the operation mode and topology of the power system change significantly, leading to large differences in data distribution between the new scenarios and the TSA database, the results of the prediction model applied online are no longer reliable and the model needs to be updated promptly. In present studies, the prediction models are often updated using transfer learning [25,26,27,28], which is widely divided into model transfer and sample transfer. In studies related to sample transfer [26,27], the model is re-trained by combining some or all of the data in the TSA database with the data in a new scenario. Although the updated model can meet the prediction requirements of both old and new scenarios, its update speed and update cost keep increasing with the emergence of new scenarios, making it difficult to meet the requirements of long-term applications. For model transfer, the model is fine-tuned using the new scenario data [29]. This method yielded a fast and exact prediction model for the new scenario, but the performance of the old scenario dropped significantly, i.e., the model suffered from a catastrophic forgetting problem. The complex time-varying feature of the system requires frequent updates to the prediction model, so this update is skipped. The rising of continual learning in recent years [30,31,32] provides three ideas to address the problem of catastrophic forgetting when models are updated. It includes a replay-based approach, a regularization-based approach, and a parameter isolation-based approach. Among them, regularization-based methods are the most elegant ones in continual learning. Instead of requiring additional storage space or increasing the model parameters, it simply adds some regularization terms to avoid excessive updates of parameters that are more relevant to the old task, enabling the model to maintain good performance in both old and new scenarios.

Based on the above analysis, Sliced Cramér Preservation (SCP) [33], a continual learning algorithm based on distribution regularization, is introduced in this paper. Combined with SCP the updated TSA model can maintain excellent performance in both old and new scenarios. With the operational changes in the system, the TSA model can constantly improve the rate of new scenarios covered by the system. In addition, this paper uses the DRSN [34] model as a classifier so that it can overcome the interference of noise and make the model more robust. The main contributions of this paper are as follows:

- Introducing the continual learning algorithm SCP to resolve the problem of catastrophic forgetting when the model is updated. It can guarantee the evaluation requirements of all scenarios at the same time in the limited data range.
- A deep residual shrinkage network is used as a classifier to reduce the impact of noise on the model learning distribution and ensure the learning ability of the model.
- The focal loss function is introduced to solve the problems caused by unbalanced training samples and hard and easy samples during the training process.

The rest of the paper is organized as follows. Section 2 describes the transient stability problem and the proposed method. Section 3 introduces the classifier model DRSN and the continual learning algorithm SCP. Section 4 describes the implementation of the SCP-DRSN model, including its evaluation metrics, loss function, and evaluation process. Section 5 presents the case studies. Section 6 discusses the proposed method. Section 7 concludes the whole paper.

## 2. Problem Formulation and Proposed Method

### 2.1. Transient Stability Assessment Problem

The power system would be subject to various large disturbances during operation, when the disturbed system can transition to a new stable operating state or return to the original state after the transient process, the system is transient stable, otherwise the system will occur transient instability [35]. The essence of transient stability assessment is to find a boundary that divides the stability and instability of a system. The data-driven method treats the power system transient stability assessment as a two-classification problem. A prediction model is constructed using operational data that reflect the transient stability information of the power system with the corresponding stability findings trained. In this study, the input X of the prediction model consists of all bus voltage magnitudes and phase angles in the system, and the output of the model is the category of transient stability. The stability classes corresponding to the samples in the training data are labeled according to the transient stability index [36]:

(1) $$\eta =\frac{360^{{}^{\circ}}-\left|\Delta \delta_{\max}\right|}{360^{{}^{\circ}}+\left|\Delta \delta_{\max}\right|},$$

where $\Delta \delta_{\max}$ is defined as the maximum relative power angle difference between any two generators during the simulation time. If η > 0, the system is transient stable and the sample is labeled as 0. Otherwise, the system is transient unstable, and the sample is labeled as 1.

### 2.2. Proposed Method

**Figure 1:**
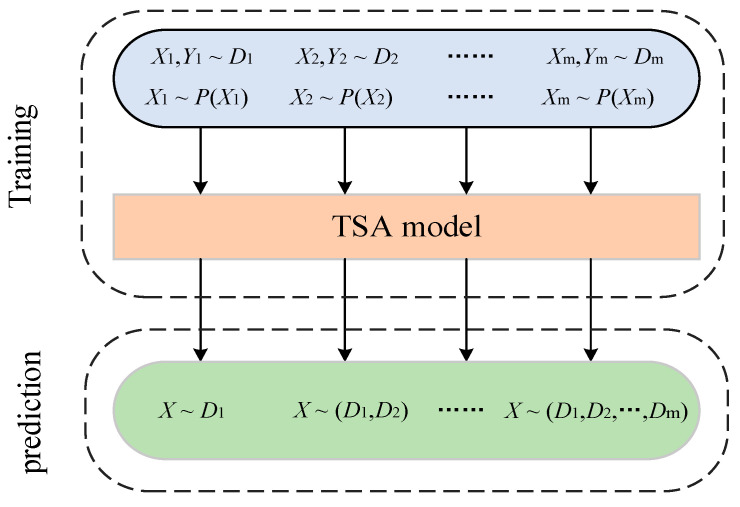
A continual learning scheme.

The input distribution of the TSA model applied online is a dynamically changing data stream owing to the constantly changing manner in which the system of maintenance and control measures operates. Let the dataset under the *i*th scenario be denoted as $D_{i}=\left\{X_{i},Y_{i}\right\}$, where $X_{i}=\left\{V_{i},\phi_{i}\right\}$ is the set of input features in the state of scenario *i*, $Y_{i}\in \left\{0,1\right\}$ corresponds to the label of its class, and $X_{i}$ follows a probability distribution of. The input data obtained in different scenarios all belong to the same task, so the class labels remain unchanged and they are denoted as $\left\{Y_{i}\right\}=\left\{Y_{i+1}\right\}$. The probability distribution of the data in different scenarios changes, denoted as $P\left(X_{i}\right)!=P\left(X_{i+1}\right)$. Nevertheless, deep models are commonly trained on static homogeneously distributed data and cannot adapt or extend their behavior as the external environment changes. Hence, a continual learning scheme is proposed, which is shown in Figure 1. The TSA model constructed in combination with continual learning can sustainably integrate and optimize the learned knowledge from non-smooth data distribution over time.

## 3. Algorithms

This section will introduce the two learning algorithms included in the proposed method, including a deep residual shrinkage network and continual learning SCP. The proposed algorithms are described in detail as follows.

### 3.1. Deep Residual Shrinkage Network

The deep residual shrinkage network is a modified network based on the residual network (DRN) [37], which is a feature learning method for strong noise or highly redundant data. It is mainly founded on three components: deep residual network, soft threshold function, and attention mechanism. Among them, the deep residual network is a modified convolutional neural network, and “shrinkage” refers to “soft thresholding”, which is a key procedure in the signal noise reduction algorithm. In the deep residual shrinkage network, the threshold required for soft thresholding is automatically set by the attention mechanism.

#### 3.1.1. Deep Residual Network

**Figure 2:**
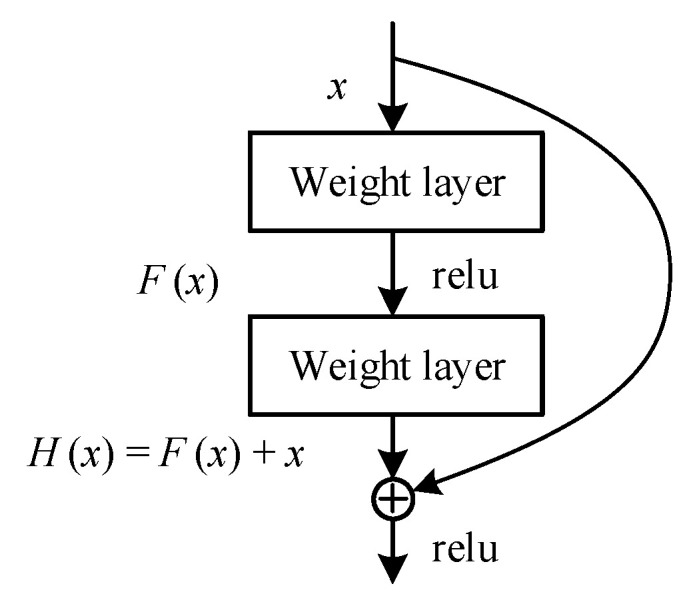
The basic blocks of residual networks.

Compared with the regular convolutional neural network, the deep residual network adopts the connection of constant paths with cross layers, and its structure is shown in Figure 2. With this path, information is transmitted more smoothly and efficiently both forward and backward. When forward computing the loss, the input signals can be propagated directly from any lower layer to the higher layer, solving the degradation problem of the deep layer network. When backward updating the gradient, the parameter gradient of the deep structure in the neural network can be transmitted to the input layer faster, resolving the gradient disappearance or explosion problem and reducing the training difficulty of the deep network.

When $F\left(x\right)=0$, then $H\left(x\right)=x$, which is a constant mapping.

#### 3.1.2. Soft Thresholding

Based on the residual unit, the residual shrinkage unit inserts the soft thresholding into the structural unit as a non-linear transformation layer. The input signal is mapped into a range by the learning activities of the neural network layers, and numbers close to 0 in that range are considered to be less important. Therefore, soft thresholding means that the feature close to 0 is changed to 0, which results in reducing the noise. The soft thresholding function is as follows:

(2) $$y=\left\{\begin{array}{cc} x-\tau & x>\tau \\ 0 & -\tau \leq x\leq \tau \\ x+\tau & x<-\tau \end{array}\right.,$$

Where x is the input, y is the output and τ is the threshold. The derivative of the soft thresholding function is formulated as follows:

(3) $$\frac{\partial y}{\partial x}=\left\{\begin{array}{cc} 1 & x>\tau \\ 0 & -\tau \leq x\leq \tau \\ 1 & x<-\tau \end{array}\right.,$$

It can be seen that the derivative values of the soft thresholding function are only 0 and 1. This property is the same as that of the ReLu activation function. Therefore, the soft thresholding function is also beneficial to prevent gradient disappearance and gradient explosion.

#### 3.1.3. Attentional Mechanism

**Figure 3:**
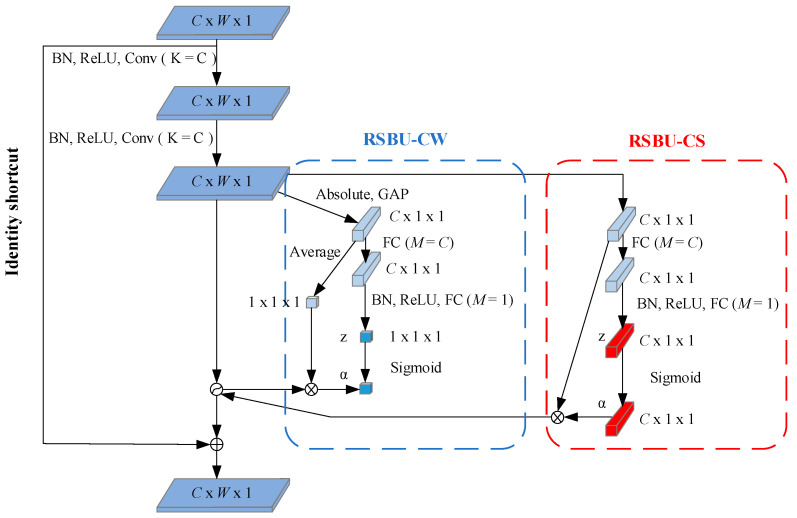
Structure diagram of modules RSBU-CW and RSBU-CS.

In practical situations, the redundant information content is usually different for different samples. The attention mechanism adaptively sets different thresholds for each sample, focusing attention on locally critical information. The deep residual shrinkage network adaptively sets thresholds by a small sub-network. Dividing the residual shrinkage module into a Residual Shrinkage Building Unit with Channel-shared thresholds (RSBU-CS) and a Residual Shrinkage Building Unit with Channel-wise thresholds (RSBU-CW), and the module structures of both are shown in Figure 3.

The threshold value of RSBU-CS is a scalar, which is applied to all channels of the feature map. The formula for calculating the threshold value for this module is as follows:

(4) $$\tau =\alpha \cdot \underset{i,j,c}{\mathrm{average}}\left|x_{i,j,c}\right|,$$

The threshold of RSBU-CW is a vector, which means that each channel of the feature map corresponds to a shrinkage threshold. The formula for calculating the threshold value of this module is as follows:

(5) $$\tau_{c}=\alpha_{c}\cdot \underset{i,j}{\mathrm{average}\left|x_{i,j,c}\right|},$$

where τ is the threshold value and α is the scaling factor. i, j and c are the indexes of width, height, and channel of the feature map X, respectively.

### 3.2. Continual Learning of SCP

#### 3.2.1. Continual Learning

**Figure 4:**
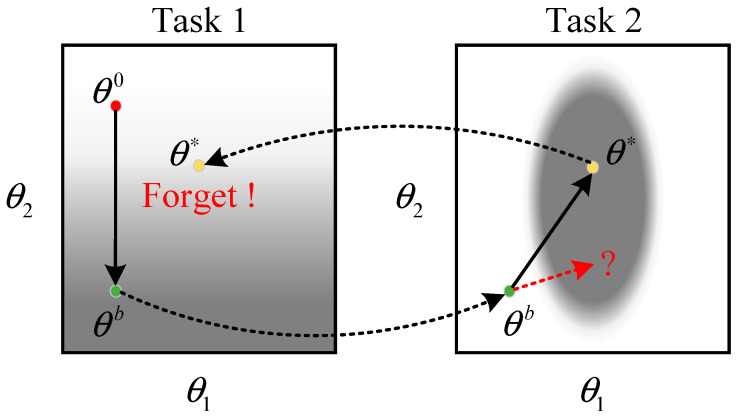
The error surfaces in tasks 1 and 2.

The main difficulty in achieving continual learning on deep neural networks is overcoming the problem of catastrophic forgetting when models are updated. The knowledge and features learned by the neural network are stored in the model parameters (e.g., convolutional kernel parameters). When a neural network learns a new task, its parameters are updated, and the knowledge of the old task is overwritten, resulting in a “catastrophic drop” in the performance of the updated model on the old task. The process is depicted in Figure 4.

In the above figure, the darker gray color corresponds to a smaller loss. The best parameter obtained by the model in Task 1 is $\theta^{b}$. When faced with Task 2, it is trained directly based on the previous task, and the parameter $\theta^{b}$ is updated $\theta^{*}$. In this case, the $\theta^{*}$ is a set of poorly behaved parameters when the model returns to task 1 again. If $\theta^{b}$ is chasing the optimum on Task 2 only updates to $\theta_{1}$ on the horizontal axis are considered and changes to $\theta_{2}$ on the vertical axis are restricted as much as possible. Then it is possible to obtain a set of parameters that perform well on both tasks.

The regularization-based continual learning algorithm adds the regularization term to the loss function of a new task to limit the variation of each weight parameter of the model to protect the old knowledge from being overwritten by the new knowledge. The loss function of the new task is shown below:

(6) $$L^{\prime }\left(\theta \right)=L\left(\theta \right)+\lambda {\sum_{i}b_{i}\left(\theta_{i}-\theta_{i}^{b}\right)}^{2},$$

where λ is the regularization factor, $L\left(\theta \right)$ denotes the original loss function of the model, and the summation term that follows is the regularization term. $\theta_{i}$ is the ith parameter of the model, and $\theta_{i}^{b}$ is the ith parameter of the model learned in the old task. The regularization factor $b_{i}$ represents the importance of the ith parameter to the old task. The larger $b_{i}$ is, the more important the ith parameter is, and the less $\theta_{i}$ can depart too far from $\theta_{i}^{b}$.

#### 3.2.2. SCP

The SCP [33] introduced in this paper is a continual learning algorithm based on distribution regularization. Compared with previous methods of sample regularization, SCP imposes less constraints on the network and can better utilize the learning ability of the network. When learning a new task, the SCP uses the slice Cramér distance in order to determine the importance of the model parameters, obtaining a matrix representing the importance. The distribution of any layer in the neural network over the previous task can be preserved by the relevant importance parameters determined, thus enabling the inheritance of the knowledge learned on the previous task. When task A is learned, the loss function of SCP on the new task B is expressed as follows:

(7) $$\underset{\theta }{\arg\min{\tilde{L}}^{B}}\left(\theta \right)=\underset{\theta }{\arg\min L^{B}}\left(\theta \right)+\lambda \sum_{m=1}^{M}{\left[\Gamma \right]}_{m,m}{\left[\theta -\theta_{A}^{*}\right]}_{m}^{2},$$

In order to extend to sequential learning of multiple tasks and to keep the memory requirements of the methods constant, the SCP follows the same framework as EWC++ [38]. The sliced-Cramér regularizer of task (t + 1) is obtained by $\Gamma_{\theta }^{\left(t\right)}=\alpha \Gamma_{\theta_{t}^{*}}+\left(1-\alpha \right)\Gamma_{\theta }^{\left(t-1\right)}$ where $\alpha \in \left[0,1\right)$ is a hyperparameter representing the importance of the new task relative to the old one.

## 4. Transient Stability Assessment of Power System Based on SCP-DRSN

**Figure 5:**
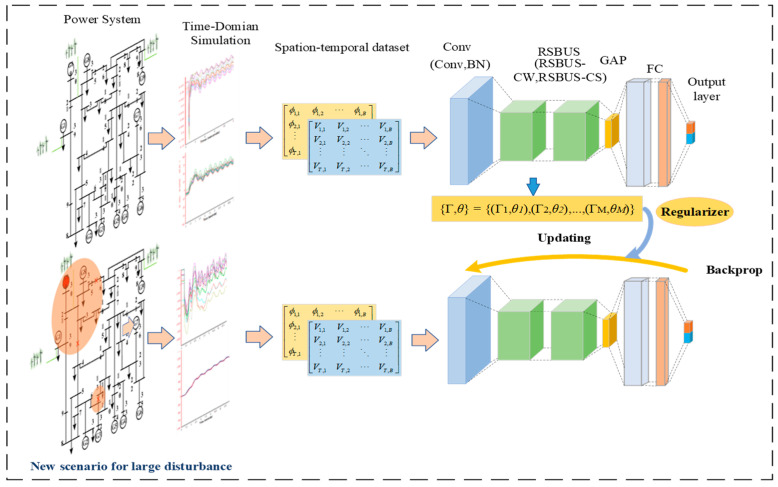
A framework based on SCP-DRSN for transient stability.

Based on the introduction in the previous section, the structure of the SCP-DRSN model proposed in this paper is shown in Figure 5. It includes an input layer, a convolutional layer, a series of residual shrinkage modules, and finally a global average pooling layer, along with a fully connected output layer, etc. In the update phase, SCP constructs regularized loss terms by computing parameter importance matrices to limit the forgetting of old knowledge while learning new data. In addition, to balance the computational complexity and model effects, two RSBU-CW modules are used near the input layer and two RSBU-CS modules are used near the output layer. The activation function used is the ReLu activation function.

### 4.1. Input Features

In this paper, the bus voltage magnitude and phase angle of the power system are chosen as the input features of the prediction model. For one, they can be obtained directly by PMU, and secondly, a large number of studies [39,40,41] have shown that they can obtain the highest precision for transient stability assessment. Based on the graphical transient features, the voltage magnitude and phase angle are constructed as two-dimensional images, respectively, and stacked on the channels to form a three-dimensional input feature $X^{2\times T\times B}$ for two channels. In summary, for all samples, the input feature is $X=\left\{V,\phi \right\}$:

(8) $$V=\left[\begin{array}{cccc} V_{1,1} & V_{1,2} & \cdots & V_{1,B}\\ V_{2,1} & V_{2,2} & \cdots & V_{2,B}\\ \vdots & \vdots & \ddots & \vdots \\ V_{T,1} & V_{T,2} & \cdots & V_{T,B} \end{array}\right],$$

(9) $$\phi =\left[\begin{array}{cccc} \phi_{1,1} & \phi_{1,2} & \cdots & \phi_{1,B}\\ \phi_{2,1} & \phi_{2,2} & \cdots & \phi_{2,B}\\ \vdots & \vdots & \ddots & \vdots \\ \phi_{T,1} & \phi_{T,2} & \cdots & \phi_{T,B} \end{array}\right],$$

### 4.2. Evaluation Indicators

In the TSA of power systems, there are characteristics of imbalance of sample labels and different costs caused by the omission of unstable samples and the false judgment of stable samples. In order to comprehensively evaluate the model performance, this paper chooses the following three evaluation metrics: Accuracy (*Acc*) rate, Misdetection (*Mis*) rate, and False-alarm (*Fal*) rate. The specific formulation is as follows:

(10) $$\begin{array}{c} Acc=\frac{TN+TP}{TN+TP+FN+FP}\\ Mis=FN/\left(FN+TP\right)\\ Fal=FP/\left(FP+TN\right), \end{array}$$

where TP and TN denote the numbers of unstable samples and stable samples with correct predictions, respectively; FP and FN denote the number of stable samples and unstable samples with incorrect predictions, respectively.

### 4.3. Focal Loss Function

In order to solve the problems of sample imbalance and serious consequences of sample misclassification in transient stability assessment, a focal loss function [42] is introduced in this paper to guide the model training. It can not only adjust the weights of positive and negative samples, but also control the weights of difficult and easy-to-classify samples. The expression is as follows:

(11) $$FL\left(Y,\hat{Y}\right)=\left\{\begin{array}{cc} -\alpha \cdot {\left(1-{\hat{y}}_{i}\right)}^{\gamma }\cdot \log{\hat{y}}_{i} & y_{i}=1\\ -\left(1-\alpha \right)\cdot {\hat{y}}_{i}{}^{\gamma }\cdot \log\left(1-{\hat{y}}_{i}\right) & y_{i}=0 \end{array}\right.,$$

where y is the real label of the sample and $\hat{y}$ is the predicted probability of the sample label. $\alpha \in \left[0,1\right]$ is a balancing factor to balance the disproportionality of positive and negative samples. γ > 0 as a modulation factor, allowing for the model to focus more on predicting difficult samples that perform badly. In this paper, we set α = 0.75 and γ = 2 through extensive simulation experiments.

### 4.4. Evaluation Process

**Figure 6:**
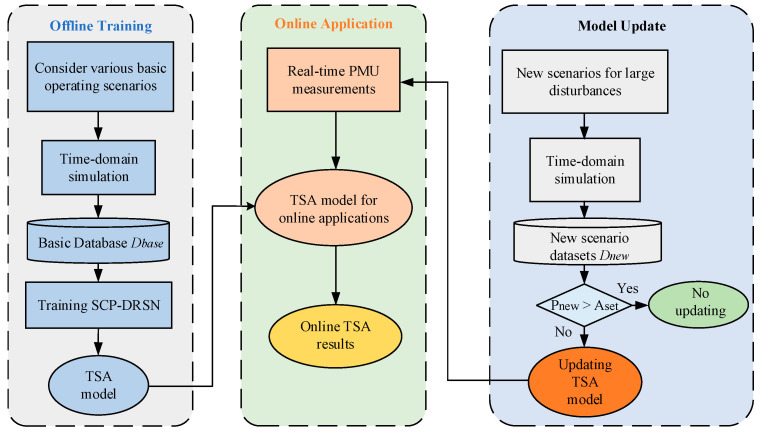
Flowchart of the proposed method.

The flow chart of the proposed method is shown in Figure 6, which consists of three parts: offline training, online application, and model update.

During the offline training process, to avoid frequent updates of the TSA model in later use, various basic operating conditions of the system should be considered as comprehensively as possible when constructing the initial TSA database. The required basic dataset is generated by the time-domain simulation software, and the model is trained based on this database.

For the online application, the operational data of the power system are collected in real-time through the PMUs. The data are processed into the structure required by the model and input into the TSA model, and the real-time evaluation results are quickly and accurately derived using the TSA model applied online.

For the model update phase, which is the focus of this paper, the power system operating conditions will change due to economic dispatch, maintenance, and other needs, and the offline initial database cannot cover all operating situations. In general, power companies can gain a list of potential operating events for the power system through forecasting. When a new operating situation emerges that was not considered before, the corresponding new scenario dataset $D_{new}$ is obtained by time domain simulation software and then the prediction precision $P_{new}$ of the TSA model is tested. When the test results are below a predetermined threshold $A_{set}$, the model is updated in time with the new scenario dataset $D_{new}$. As the model update process is executed, the probability of encountering unknown operating situations is gradually reduced and the generalization capability of the model is gradually improved.

## 5. Case Study

The proposed method was tested on the modified New England 10-machine 39-bus system and the IEEE 118-bus system. The TSA model of this paper is implemented in the Pytorch environment, and the programming language is Python. The computer is configured with an Intel(R) Core (TM) i5-10200H 2.40 GHz CPU and 16.0 GB RAM.

### 5.1. A Modified New England 39-Bus System

#### 5.1.1. Dataset Generation

The New England 10-machine 39-bus system contains 10 generators, 39 buses, and 46 transmission lines. The standard example is modified in this paper by connecting the wind farms at buses 2, 29, and 39, respectively. This paper applies the python API of the simulation software PSS/E to implement batch transient simulation and generate three scenarios with different distributions of datasets required for the tests. The generator is set to the GENROU model, the load is set to the constant impedance model, the simulation step is set to 0.01 s, and the sampling frequency is set to 100 Hz. The bus voltage amplitudes and phase angles of the 5 cycles after fault disconnection are selected as the initial input features, and the data are labeled using the stability criterion. The above labeled sample dataset is divided into the training set, test set, and validation set according to the ratio of 8:1:1. Among them, the training set is used for the training of the model, the validation set is used for the selection of model hyperparameters, and the test set is used to test the performance of the model.

The three typical scenarios mentioned above are scenario 1 (which covers as many operating conditions of the system as possible), scenario 2 (which simulates the system generation and dispatch with a major change in tide), and scenario 3 (which reflects a huge change in the system topology). The three typical scenarios simulation generated datasets correspond to the basic dataset $D_{base}$, the new scenario dataset $D_{new1}$, and the new scenario dataset $D_{new2}$, respectively. The simulation settings are as follows.

The dataset $D_{base}$ for scenario 1: considering that the new energy penetration rate changes in steps of 5% to 20% and the system load level changes in steps of 5% from 75% to 125%. A three-phase short-circuit fault is set for 34 non-transformer branches, and the fault time duration is set to 0.02~0.2 swith a step of 0.02 s. The simulation generates a total of 14,960 samples. Among them, the number of stable samples is 8450 and the number of unstable samples is 6510.

In order to avoid an excessive number of simulation samples, the new scenarios dataset is appropriately reduced. Considering that the new energy penetration rate changes within 10% to 20% in steps of 5%, the load level changes within 80% to 120% in steps of 5%, and the fault time duration is 0.04 to 0.2 s in steps of 0.04 s.

**Figure 7:**
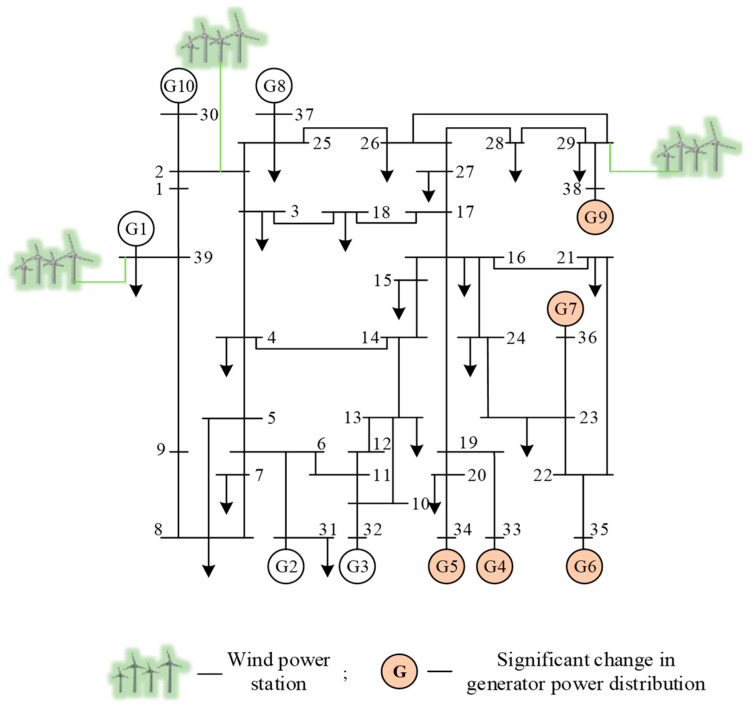
Topology of scenario 2 of the modified New England 39-bus system.

The dataset $D_{new1}$ for scenario 2: significantly changes the generator terminal probability distribution, the topology of this scenario is shown in Figure 7. Three-phase short-circuits faults are set for 34 non-transformer branches. The simulation generates a total of 4590 samples. Among them, the number of stable samples is 3018 and the number of unstable samples is 1572.

**Figure 8:**
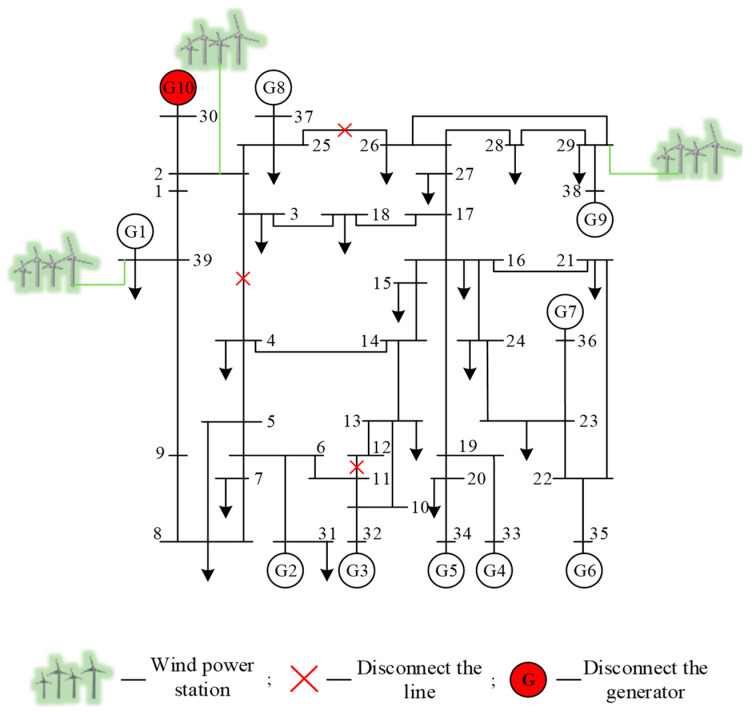
Topology of scenario 3 of the modified New England 39-bus system.

The dataset $D_{new2}$ for scenario 3: disconnecting a transformer branch and two non-transformer branches and removing one generator, the topology of this scenario is shown in Figure 8. A three-phase short-circuit fault is implemented for the remaining 32 non-transformer branches. The simulation generates 5280 samples, of which 3171 are stable samples and 2109 are unstable samples.

#### 5.1.2. Comparison with Other Models

In order to verify the superiority of the basic model of the method in this paper, the DRSN is compared and validated with the commonly used machine learning methods RF, SVM, and deep learning methods MLP, CNN, and DRN on the scenario 1 dataset $D_{base}$. The results are shown in Table 1. Note that in order to test the effectiveness of the focal loss function, the normal cross-entropy loss function is used except for the method proposed in this paper. The parameters of the neural network are initialized using the Xavier function, an optimization algorithm Adam can adaptively adjust the learning rate to accelerate model convergence, and a dropout regularization technique is used to avoid model overfitting.

Obviously, the shallow machine learning models RF and SVM perform poorer than the deep learning model in all metrics, in which the *Mis* reaches 3.45% and 3.30%, respectively. Although the *Acc* of MLP, CNN, and DRN in deep learning is improving sequentially, *Mis* and *Fal* still remain relatively high due to the problem of sample imbalance is not considered. The DRSN is an improved network on the DRN, with the effect of the shrinkage module and the focal loss function, *Acc* is increased by 0.68%, and *Mis* and *Fal* are reduced by 1.37% and 0.39%, respectively. Compared with the regular CNN, *Acc* improves by 0.95%, *Mis* and *Fal* decrease by 2.31% and 0.62%, respectively.

#### 5.1.3. Testing the Generalizability of the Model in New Scenarios with Large Disturbances

**Figure 9:**
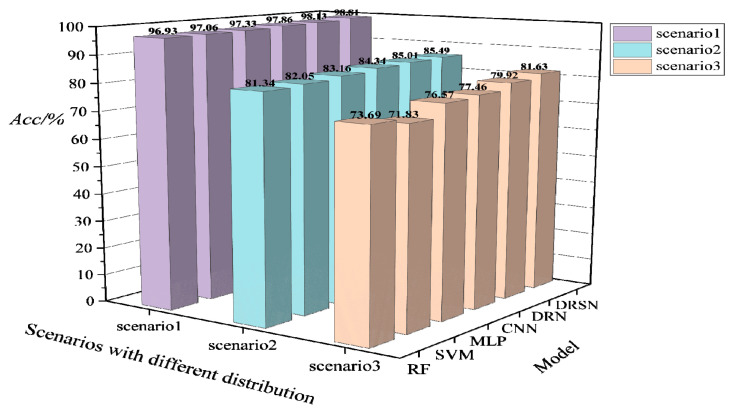
*Acc* performance of different models trained on the modified New England 39-bus system based on the fixed distribution for the new scenarios with large disturbances.

In the practical operation of the system, new scenarios with huge changes in topology and power distribution may be encountered. In order to test the generalizability of the models in such scenarios, the models are trained based on the scenario 1 dataset $D_{base}$, and then tested on the scenario 2 dataset $D_{new1}$ and the scenario 3 dataset $D_{new2}$, respectively, after the training is completed. The test result is shown in Figure 9.

It can be seen from Figure 9 that the *Acc* performance of each model in the new scenario shows a clear decrease. Likewise, the deep learning model has a lighter decline than the machine learning model, but the prediction accuracy no longer meets the requirements. The *Acc* of the models drops about 15% on average under scenario 1, and the Acc of the models drops about 20% on average under scenario 2. The analysis of the test results indicates that the data distribution of the run data generated in the two new scenarios is already significantly different from the initial basic data. Especially in the face of scenario 3, the model almost loses its effectiveness for TSA. Therefore, the model needs to be updated in advance before such scenarios occur.

#### 5.1.4. Comparison of Different Update Schemes

**Figure 10:**
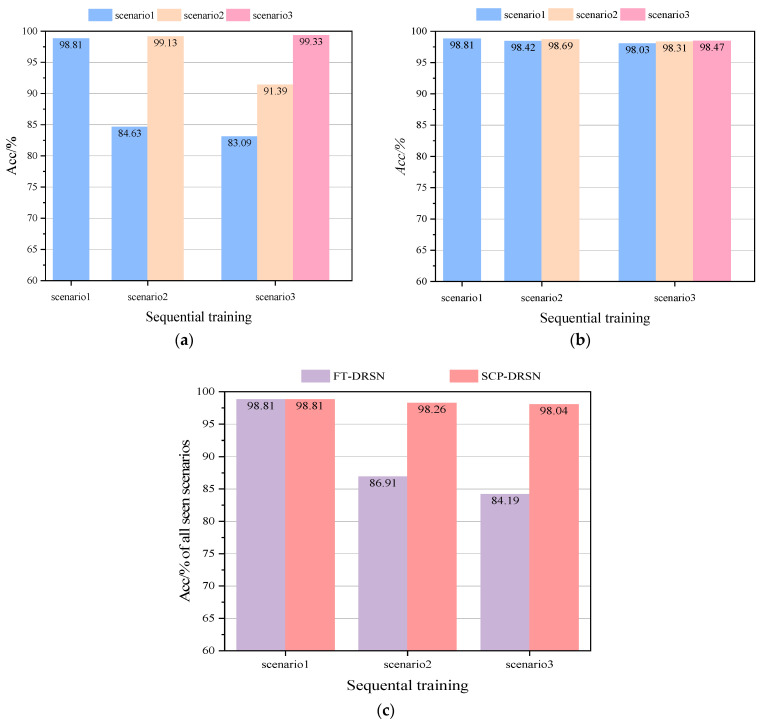
*Acc* comparison of two different update schemes on the modified New England 39-bus system: (**a**) *Acc* comparison of FT-DRSN in different scenarios, (**b**) *Acc* comparison of SCP-DRSN in different scenarios, and (**c**) *Acc* comparison in all seen scenarios.

In order to demonstrate the superiority of the update scheme proposed in this paper, the update effects of two different update schemes, fine-tuning (FT) in transfer learning and continual learning SCP, are tested separately under the condition that only the new scenario dataset is used for updating. The training and updating process of model DRSN is as follows. Firstly, the training is completed on the basic dataset $D_{base}$ of scenario 1, and then the training is updated on scenario 2 and scenario 3 in turn by following two different updating schemes. After each training update mentioned above is completed, the model is tested on the current scenario as well as on the past scenarios, and then tested on joint all seen scenarios. The test results are shown in Figure 10.

According to Figure 10a, the Finetuning-DRSN model constructed by the fine-tuning update scheme can have 99.13% and 99.33% accuracy in the new scenario, but in the previous scenarios, it only has 84.63% and 83.09% accuracy, with a catastrophic drop in performance. Figure 10b shows that the accuracy of the SCP-DRSN model constructed under the continual learning update scheme is still maintained at 97.65%, 98.31%, and 98.47% for each scenario after the third scenario training is completed. It verified the ability of the model to continuously learn under this method. The performance of the model on all seen scenarios is shown in Figure 10c. The test results show that the TSA model combined with the continual learning update scheme can maintain a high level and smooth performance during the emergence of new scenarios, and the coverage of the model for new scenarios is constantly improving.

#### 5.1.5. Robustness Analysis

In practical applications, PMU measurements are influenced by noises. In order to test the robustness of the model to noise, Gaussian white noises with signal-to-noise (SNRs) of 40 dB, 30 dB, and 20 dB were added to the original test data. The test results are shown in Table 2.

It is observable from Table 2 that the prediction performance of each model decreases to some extent with the increase of noise. When the SNR is 20 dB, in terms of *Acc*, the model RF, SVM, and MLP decrease by 2.22%, 2.54%, and 2.01%, respectively. The models CNN, DRN, and DRSN decrease by 1.78%,1.44%, and 0.76%, respectively. Compared to DRN, DRSN has 0.68% fewer in *Acc*. For *Mis*, RF is up to 5.81% at its peak, which is an increase of 2.36% compared to non-noise. The *Fal* of SVM is 6.98% at its peak, an increase of 4.3%. Due to the noise immunity of DRSN, the *Fal* and *Mis* of the model are only 2.24% and 0.71% under the most severe conditions of the test noise. Compared with the test results of DRN, the anti-noise effect is obvious.

### 5.2. A Lager Test SYSTEM

In order to verify the effectiveness of the proposed TSA method in large-scale power systems, the SCP-DRSN-based TSA framework is applied to the IEEE 118-bus system.

#### 5.2.1. Dataset Generation

The IEEE 118-bus system consists of 19 generators, 35 synchronous capacitors, 177 transmission lines, 9 transformers, and 91 loads. In the same manner, three scenario datasets with different distributions are generated with PSS\E. Scenario 1 has 18,588 samples, including 11,025 stable samples and 7563 unstable samples. Scenario 2 has 8910 samples, of which 5009 are stable samples and 3910 are unstable. Scenario 3 has 4150 samples, of which 1795 are stable samples and 2355 are unstable.

#### 5.2.2. Model Performance Analysis

**Figure 11:**
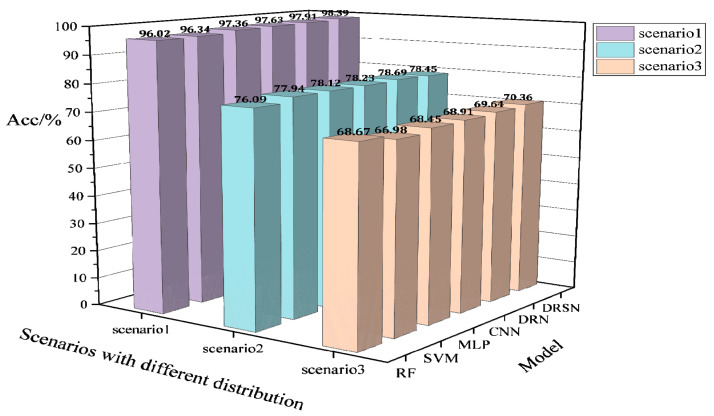
*Acc* performance of different models trained on the IEEE 118-bus system based on the fixed distribution for a new scenario with large disturbances.

**Figure 12:**
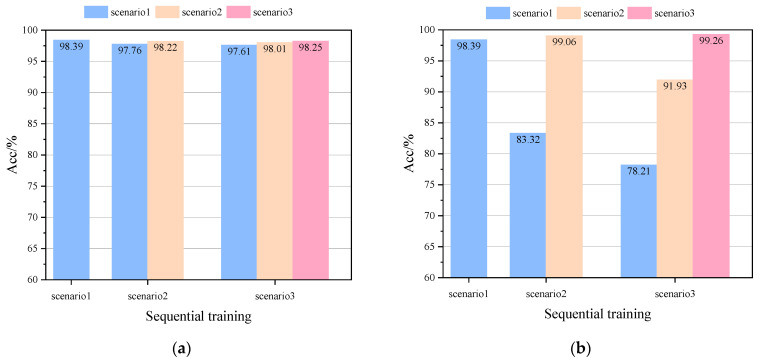

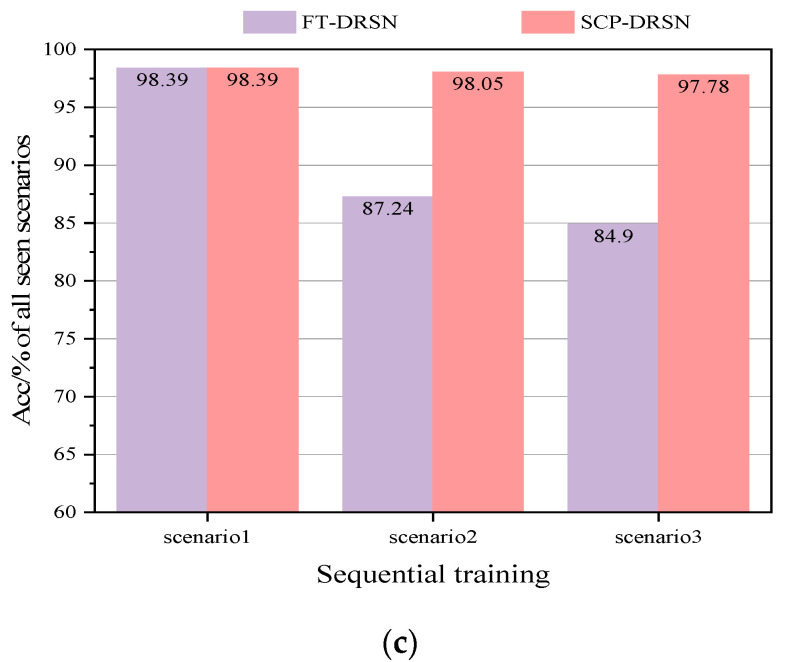
*Acc* comparison of two different update schemes on the IEEE 118-bus system: (**a**) *Acc* comparison of FT-DRSN in different scenarios, (**b**) *Acc* comparison of SCP-DRSN in different scenarios, and (**c**) *Acc* comparison in all seen scenarios.

The test procedure and configuration in this section are the same as in the aforementioned case for the modified New England 39-bus system. The results of comparing the basic model DRSN with other models on the IEEE 118-bus system are shown in Table 3, and the results of robustness tests in the PMU noise environment are shown in Table 4. The generalizability test of the model on the new scenarios of IEEE 118-bus is shown in Figure 11. The effects of two different updates of fine-tuning (FT) and continual learning are shown in Figure 12.

## 6. Discussion

In order to solve the update problem caused by frequent changes in the topology and operation of the power system to the TSA model, this paper introduces the Sliced Cramér Preservation (SCP) algorithm of continual learning in order to perform the update operation of the model. For the proposed SCP-DRSN model, through the experimental results we can find that DRSN has stronger data mining and anti-noise learning ability compared to other machine learning and deep learning algorithms in terms of classifier selection. Meanwhile, with the focal loss function, the test results in the two provided cases clearly show that DRSN has the optimal performance on the metrics *Acc*, *Mis,* and *Fal*.

The test results on the generated datasets with three different distributions confirm the necessity of TSA model updates. The study compared the experimental results of the two update schemes under the condition of using only new scenario data updates. The fine-tuned model can just meet the assessment requirements of the current scenario, and the model needs to be updated or switched frequently across scenarios. For the continual learning update scheme proposed in this paper, the assessment capability of the model is effectively supplemented with the emergence of new scenarios, and the updated model can cover all operational scenarios. This characteristic makes the method advantageous in terms of data storage. From the test results, the accuracy of the model under the continual learning update scheme shows a certain degree of fluctuation across scenarios, and this fluctuation results from the means of implementation of the regularized continual learning algorithm. The SCP ensures that the model performs in all scenarios by adjusting the regularization factor λ and the relative importance parameter α for the new and old tasks. If the model sets λ and α too high to maintain the performance in the old scenarios, the model parameters will not be updated effectively. If the model only pursues high accuracy in the new scenario by setting λ and α too small, the updated parameters will lose applicability to the old scenarios. However, when such fluctuations exceed the allowed limits, it means that the model with a fixed number of parameters has reached its capacity limit. The capacity of the TSA model for continual learning can be maintained by increasing the number of parameters based on the original size.

## 7. Conclusions

A TSA model combined with a continual learning update scheme is proposed for the situation where the accuracy of the prediction model is not satisfying the requirements due to system changes under large disturbances. Continual learning solves the problem of catastrophic forgetting during model updates in comparison to updates by fine-tuning methods in transfer learning. It retains the knowledge learned by the model in previous scenarios and that it is a scalable method for updating the model. In studies of the modified New England 39-bus system and the IEEE 118-bus system, it was shown that the framework requires updating of the model only using the new scenario dataset and that the updated model meets the assessment requirements under both old and new scenarios. As the updates proceeded, the model coverage of the system operation scenarios also increased.

In future work, continual learning will be of great significance for building models with multiple assessment capabilities. For example, a model with the capability of transient stability assessment, frequency stability assessment, and voltage stability assessment at the same time.

## Figures and Tables

**Table 1:** Performance of different models on modified IEEE 39 bus system.

Model	*Acc*/%	*Mis*/%	*Fal*/%
RF	96.93	3.45	2.79
SVM	97.06	3.30	2.68
MLP	97.33	2.83	2.56
CNN	97.86	2.50	2.10
DRN	98.13	1.56	1.87
DRSN	98.81	0.19	1.48

**Table 2:** Performance of the different models on the modified New England 39-bus system with different SNRs.

Model	40 dB			30 dB			20 dB		
	*Acc/%*	*Mis/%*	*Fal/%*	*Acc/%*	*Mis/%*	*Fal/%*	*Acc/%*	*Mis/%*	*Fal/%*
RF	96.72	3.61	3.07	96.01	4.08	3.61	94.71	5.81	4.89
SVM	96.93	2.98	3.14	96.27	3.51	3.73	94.39	4.41	6.98
MLP	97.13	2.20	3.38	96.46	2.82	4.07	95.12	3.77	5.70
CNN	97.66	2.57	3.29	97.03	2.75	4.02	95.88	3.29	4.85
DRN	98.06	1.75	2.19	97.59	2.19	3.61	96.62	3.11	4.22
DRSN	98.79	0.21	1.45	98.48	0.38	1.75	98.03	0.71	2.24

**Table 3:** Performance of different models on the IEEE 118-bus system.

Model	*Acc*/%	*Mis*/%	*Fal*/%
RF	96.02	2.34	5.36
SVM	96.34	2.62	3.68
MLP	97.36	2.04	3.48
CNN	97.63	2.01	3.39
DRN	97.91	1.86	2.91
DRSN	98.39	0.45	1.74

**Table 4:** Performance of different models on the IEEE 118-bus system with different SNRs.

Model	40 dB			30 dB			20 dB		
	*Acc/%*	*Mis/%*	*Fal/%*	*Acc/%*	*Mis/%*	*Fal/%*	*Acc/%*	*Mis/%*	*Fal/%*
RF	95.91	2.35	5.48	95.54	2.62	7.76	94.72	4.17	7.36
SVM	96.28	3.50	3.88	96.07	2.66	4.95	95.21	1.57	7.73
MLP	97.25	2.21	3.51	96.74	2.91	5.14	95.69	4.71	7.42
CNN	97.52	2.17	3.49	96.88	2.86	5.04	95.80	4.87	7.17
DRN	97.84	2.06	3.02	97.09	2.76	4.66	95.96	4.83	6.86
DRSN	98.22	0.56	1.76	98.17	0.69	1.94	98.03	0.93	2.33

## Data Availability

Not applicable.

## References

[1] Du E.S., Zhang N., Hodge B.M., Wang Q., Kang C.Q., Kroposki B., Xia Q. (2018). The Role of Concentrating Solar Power Toward High Renewable Energy Penetrated Power Systems. IEEE Trans. Power Syst..

[2] Huy N.D., Huy C.D., Chien N.D., Viet N.X.H. (2015). Simulation of a Power Grid Blackout Event in Vietnam. Proceedings of the General Meeting of the IEEE-Power-and-Energy-Society.

[3] Yan R.F., Nahid Al M., Saha T.K., Bai F.F., Gu H.J. (2018). The Anatomy of the 2016 South Australia Blackout: A Catastrophic Event in a High Renewable Network. IEEE Trans. Power Syst..

[4] Xue Y.S., Xiao S.J. (2013). Generalized congestion of power systems: Insights from the massive blackouts in India. J. Mod. Power Syst. Clean Energy.

[5] Hashiesh F., Mostafa H.E., Khatib A.R., Helal I., Mansour M.M. (2012). An Intelligent Wide Area Synchrophasor Based System for Predicting and Mitigating Transient Instabilities. IEEE Trans. Smart Grid.

[6] Liu Y., Sun K., Yao R., Wang B. (2019). Power System Time Domain Simulation Using a Differential Transformation Method. IEEE Trans. Power Syst..

[7] Zadkhast S., Jatskevich J., Vaahedi E. (2015). A Multi-Decomposition Approach for Accelerated Time-Domain Simulation of Transient Stability Problems. IEEE Trans. Power Syst..

[8] Bhui P., Senroy N. (2017). Real-Time Prediction and Control of Transient Stability Using Transient Energy Function. IEEE Trans. Power Syst..

[9] Xu Y., Dong Z.Y., Zhang R., Xue Y.S., Hill D.J. (2015). A Decomposition-Based Practical Approach to Transient Stability-Constrained Unit Commitment. IEEE Trans. Power Syst..

[10] Karami A. (2011). Power system transient stability margin estimation using neural networks. Int. J. Electr. Power Energy Syst..

[11] De La Ree J., Centeno V., Thorp J.S., Phadke A.G. (2010). Synchronized Phasor Measurement Applications in Power Systems. IEEE Trans. Smart Grid.

[12] Wang Y., Li W.Y., Lu J.P. (2010). Reliability Analysis of Wide-Area Measurement System. IEEE Trans. Power Deliv..

[13] Zhou Y.Z., Wu J.Y., Yu Z.H., Ji L.Y., Hao L.L. (2016). A Hierarchical Method for Transient Stability Prediction of Power Systems Using the Confidence of a SVM-Based Ensemble Classifier. Energies.

[14] Liu C.X., Sun K., Rather Z.H., Chen Z., Bak C.L., Thogersen P., Lund P. (2014). A Systematic Approach for Dynamic Security Assessment and the Corresponding Preventive Control Scheme Based on Decision Trees. IEEE Trans. Power Syst..

[15] Liu C.X., Tang F., Bak C.L. (2018). An Accurate Online Dynamic Security Assessment Scheme Based on Random Forest. Energies.

[16] Siddiqui S.A., Verma K., Niazi K.R., Fozdar M. (2018). Real-Time Monitoring of Post-Fault Scenario for Determining Generator Coherency and Transient Stability Through ANN. IEEE Trans. Ind. Appl..

[17] Zheng L., Hu W., Zhou Y.F., Min Y., Xu X.L., Wang C.M., Yu R. (2017). Deep Belief Network Based Nonlinear Representation Learning for Transient Stability Assessment. Proceedings of the 2017 IEEE Power & Energy Society General Meeting.

[18] Shi Z.T., Yao W., Zeng L.K., Wen J.F., Fang J.K., Ai X.M., Wen J.Y. (2020). Convolutional neural network-based power system transient stability assessment and instability mode prediction. Appl. Energy.

[19] Yan R., Geng G.C., Jiang Q.Y., Li Y.L. (2019). Fast Transient Stability Batch Assessment Using Cascaded Convolutional Neural Networks. IEEE Trans. Power Syst..

[20] Yu J.J.Q., Hill D.J., Lam A.Y.S., Gu J.T., Li V.O.K. (2018). Intelligent Time-Adaptive Transient Stability Assessment System. IEEE Trans. Power Syst..

[21] Zhu L.P., Hill D.J., Lu C. (2020). Hierarchical Deep Learning Machine for Power System Online Transient Stability Prediction. IEEE Trans. Power Syst..

[22] Huang J.Y., Guan L., Su Y.S., Yao H.C., Guo M.X., Zhong Z. (2020). Recurrent Graph Convolutional Network-Based Multi-Task Transient Stability Assessment Framework in Power System. IEEE Access.

[23] Luo Y.H., Lu C., Zhu L.P., Song J. (2021). Data-driven short-term voltage stability assessment based on spatial-temporal graph convolutional network. Int. J. Electr. Power Energy Syst..

[24] Ren C., Xu Y. (2019). A Fully Data-Driven Method Based on Generative Adversarial Networks for Power System Dynamic Security Assessment With Missing Data. IEEE Trans. Power Syst..

[25] Li J.M., Zhao Y., Lee Y.H., Kim S.J. (2019). Learning to Infer Voltage Stability Margin Using Transfer Learning. Proceedings of the IEEE Data Science Workshop (DSW).

[26] Cui H., Wang Q., Ye Y.J., Tang Y., Lin Z.Z. (2022). A combinational transfer learning framework for online transient stability prediction. Sustain. Energy Grids Netw..

[27] Jafarzadeh S., Moarref N., Yaslan Y., Genc V.M.I. (2019). A CNN-Based Post-Contingency Transient Stability Prediction Using Transfer Learning. Proceedings of the 11th International Conference on Electrical and Electronics Engineering (ELECO).

[28] Ren C., Xu Y. (2020). Transfer Learning-Based Power System Online Dynamic Security Assessment: Using One Model to Assess Many Unlearned Faults. IEEE Trans. Power Syst..

[29] Zhou Y.Z., Guo Q.L., Sun H.B., Yu Z.H., Wu J.Y., Hao L.L. (2019). A novel data-driven approach for transient stability prediction of power systems considering the operational variability. Int. J. Electr. Power Energy Syst..

[30] Parisi G.I., Kemker R., Part J.L., Kanan C., Wermter S. (2019). Continual lifelong learning with neural networks: A review. Neural Netw..

[31] De Lange M., Aljundi R., Masana M., Parisot S., Jia X., Leonardis A., Slabaugh G., Tuytelaars T. (2022). A Continual Learning Survey: Defying Forgetting in Classification Tasks. IEEE Trans. Pattern Anal. Mach. Intell..

[32] Li Z.Z., Hoiem D. (2016). Learning Without Forgetting. Proceedings of the 14th European Conference on Computer Vision (ECCV).

[33] Kolouri S., Ketz N.A., Soltoggio A., Pilly P.K. Sliced Cramer Synaptic Consolidation for Preserving Deeply Learned Representations. Proceedings of the International Conference on Learning Representations.

[34] Zhao M.H., Zhong S.S., Fu X.Y., Tang B.P., Pecht M. (2020). Deep Residual Shrinkage Networks for Fault Diagnosis. IEEE Trans. Ind. Inform..

[35] Hatziargyriou N., Milanovic J., Rahmann C., Ajjarapu V., Canizares C., Erlich I., Hill D., Hiskens I., Kamwa I., Pal B. (2021). Definition and Classification of Power System Stability—Revisited & Extended. IEEE Trans. Power Syst..

[36] Pavella M., Ernst D., Ruiz-Vega D. (2000). Transient Stability of Power Systems: A Unified Approach to Assessment and Control.

[37] He K.M., Zhang X.Y., Ren S.Q., Sun J. (2016). Residual Learning for Image Recognition. Proceedings of the 2016 IEEE Conference on Computer Vision and Pattern Recognition (CVPR).

[38] Schwarz J., Luketina J., Czarnecki W.M., Grabska-Barwinska A., Teh Y.W., Pascanu R., Hadsell R. (2018). Progress & Compress: A scalable framework for continual learning. Proceedings of the 35th International Conference on Machine Learning (ICML).

[39] Xu Y., Zhang R., Zhao J.H., Dong Z.Y., Wang D.H., Yang H.M., Wong K.P. (2016). Assessing Short-Term Voltage Stability of Electric Power Systems by a Hierarchical Intelligent System. IEEE Trans. Neural Netw. Learn. Syst..

[40] Gomez F.R., Rajapakse A.D., Annakkage U.D., Fernando I.T. (2011). Support Vector Machine-Based Algorithm for Post-Fault Transient Stability Status Prediction Using Synchronized Measurements. IEEE Trans. Power Syst..

[41] Rajapakse A.D., Gomez F., Nanayakkara K., Crossley P.A., Terzija V.V. (2010). Rotor Angle Instability Prediction Using Post-Disturbance Voltage Trajectories. IEEE Trans. Power Syst..

[42] Lin T.-Y., Goyal P., Girshick R., He K., Dollar P. (2020). Focal Loss for Dense Object Detection. IEEE Trans. Pattern Anal. Mach. Intell..

